# Microstructure Evolution and Mechanical Properties of Ferrite–Austenite Duplex Fe-Mn-Al-(Cu)-C Steel under Different Annealing Temperatures

**DOI:** 10.3390/ma15228271

**Published:** 2022-11-21

**Authors:** Xiang Yan, Yiming Wu, Minghe Zhang, Songsong Liu, Lihui Sun, Yunli Feng

**Affiliations:** College of Metallurgy and Energy, North China University of Science and Technology, Tangshan 063210, China

**Keywords:** medium Mn steels, intercritical annealing, microstructure, mechanical properties

## Abstract

The effect of Cu addition and the intercritical annealing (IA) temperature on the microstructural evolution and mechanical properties of Fe-0.4C-7Mn-4Al (wt%) was investigated via scanning electron microscopy (SEM), electron backscatter diffraction (EBSD), X-ray diffraction (XRD) and nanoindentation tests. The results showed that the volume fraction and the average grain size of austenite, and the fraction of high angle grain boundaries, increased with IA temperature increase in the range of 650 °C to 710 °C. The addition of Cu facilitates the formation of Cu-rich nanoparticles, raises the volume fraction of austenite, and delays the recrystallization of austenite. As IA temperature increased, the yield strength (YS), ultimate tensile strength (UTS), and Lüders bands strain (LBS) decreased in both experimental steels. The Cu addition not only increases the YS of medium Mn steel but also benefits the decrease of LBS. The best comprehensive mechanical properties were obtained at the IA temperature of 690 °C in the studied steel, with Cu addition. According to nanoindentation experiments, the Cu addition raises the hardness of ferrite and austenite from 4.7 GPa to 6.3 GPa and 7.4 GPa to 8.5 GPa, respectively, contributing to the increase of YS of medium-Mn steel.

## 1. Introduction

The lightweight automobile is an effective means to reduce exhaust emissions and environmental pollution [1,2]. To adopt a lightweight auto body design and ensure passenger safety, the third-generation Advanced High Strength Steels (AHSS) have recently drawn significant attention worldwide. Medium manganese steels with an excellent strength-ductility combination are a new category of AHSS and are regarded as the most promising weight-saving material in automobiles [1,2,3,4]. A high strength-to-weight ratio for these steels reduces automobile weight [5]. Further light-weighting becomes possible by the addition of Al in medium Mn steel by reducing the density of medium Mn steels [5,6]. Medium Mn steels consist of a dual-phase or tri-phase structure composed of metastable austenite and fine ferrite and, or δ-ferrite, depending on chemical composition and processing route. The transformation-induced plasticity (TRIP) effect and the twinning-induced plasticity (TWIP) effect of retained austenite (RA) plays a significant role in improving the mechanical properties of this class of steels. Intercritical annealing (IA) is the most efficient way to tailor the austenite volume fraction, austenite grain size, solute partitioning, and mechanical stability [7,8,9,10]. Through the optimization of intercritical annealing conditions, e.g., isothermal holding temperature and time, the amount and stability of retained austenite are tuned to enhance the TRIP and, or TWIP effect, enabling this type of steels with the excellent combination of an ultimate tensile strength (UTS) of 800–1100 MPa and a total elongation (TE) of 25–50% [9,10,11,12]. 

Previous studies involving medium Mn steels mainly focused on the Fe-C-Mn-Al system with excellent mechanical properties [13,14,15,16]. In this regard, Lee et al. reported a good combination of UTS (786 MPa) and TE (47%) in Fe-10.1Mn-6.3Al-0.26C (wt%) steel [14]. An excellent combination of UTS of 1007 MPa and TE of 65% was obtained in Fe-11Mn-4Al-0.2C (wt%) steel after intercritical annealing at 770 °C [15]. The (α + γ) duplex lightweight steel showing the operation of both TRIP and TWIP mechanisms with 77% ductility was developed of Fe–0.3C–8.5Mn–5.6Al steel [16]. In recent years, the addition of a Cu element in medium Mn steels has attracted considerable attention [17,18,19]. Song et al. investigated the microstructure and mechanical properties of duplex lightweight Fe-0.5C-12Mn-7Al-(0,3)Cu (wt%) steels under varying annealing temperatures [17]. The result showed the Cu addition not only raised the volume fraction of RA, which enhanced the combination of strength and ductility, but also delayed the recrystallization of the austenite. A Fe-0.048C-5.69Mn-1.88Cu-1.96Ni-0.42Si (wt%) steel was designed and subjected to IA and tempering [18]. The yield strength (YS) and uniform elongation (UE) significantly increased by tempering because hierarchical Cu particles increased the yield strength of ferrite by ~267 MPa. According to Yan et al. [19], the addition of Cu and Ni in medium Mn steel not only increase reversed austenite fraction but also facilitate the formation of annealing twins during the austenite reversed transformation (ART) annealing. The product of tensile strength and total elongation (PSE) above 42 GPa% was obtained in Fe-0.12C-6.2Mn-1.4Si-0.93Ni-1.4Cu (wt%) steel [19]. Overall, the exploration of Cu alloying treatment for medium Mn steels is limited to low carbon low Al content, or high carbon high Al content. However, the influence of Cu alloying on medium Mn steels with medium carbon medium Al content has not been systematically investigated. 

Therefore, it is necessary to carry out systematic and detailed studies on medium Mn steels with medium carbon medium Al content to elucidate the relationship among the microstructure, mechanical properties, and IA procedure. In this paper, the medium Mn with medium carbon medium Al content steel is designed as the research object, using the IA process to produce RA. The microstructure evolution, including the austenite volume fraction and grain size after IA at different temperatures, was characterized via scanning electron microscopy (SEM), electron backscattered diffraction (EBSD), and X-ray diffraction (XRD) technology. The mechanical properties were tested and nano-indentation experiments were carried out. The effect of Cu alloying and IA temperatures on microstructure and mechanical properties is studied and discussed to guide further developments of Cu-containing medium Mn steel.

## 2. Materials and Methods

**Figure 1:**
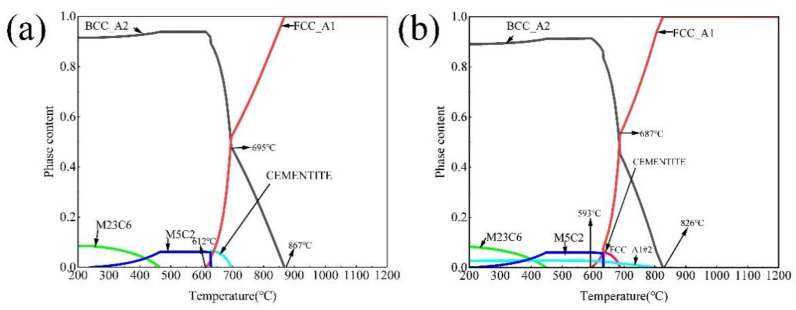
The results calculated using Thermal Calc software. (**a**) The calculated phase fraction as a function of temperature in 0Cu steel; (**b**) the calculated phase fraction as a function of temperature in 3Cu steel.

Two 45 kg experimental ingots were prepared via a vacuum induction furnace with the nominal composition of 0.4C-7Mn-4Al-0/3Cu (wt.%). The chemical composition of the cast alloys is shown in Table 1. The as-cast alloys were homogenized at 1200 °C for 5 h followed by hot forging to 25 mm thick plates. The forged plates were homogenized at 1200 °C for 2 h and hot rolled to 4 mm thick strips. The starting and finishing rolling temperatures were 1100 °C and 880 °C, respectively, followed by air cooling. The 4-mm-thick hot-rolled sheets were cold rolled to make 1.6 mm thickness sheets at room temperature. Thermo Calc equilibrium calculation was performed using the Thermocalc 2019b software with the TCFE7 database. As shown in Figure 1, the temperatures of A_C1_ and A_C3_ of the experimental steels without/with Cu addition are 621 °C and 867 °C, 593 °C and 826 °C, respectively. The Cu addition lowers the A_C1_ and A_C3_ temperatures of experimental steels. The calculated result indicates that the Cu-rich precipitates may precipitate below 805 °C. Samples of cold-rolled steels were annealed at 650 °C, 670 °C, 690 °C and 710 °C for 60 min, followed by air cooling to room temperature. The annealed sample of 0.4C-7Mn-4Al-0/3Cu steels were named 0Cu650, 0Cu670, 0Cu690, 0Cu710, 3Cu650, 3Cu670, 3Cu690 and 3Cu710, respectively. 

The microstructure was characterized by scanning electron microscope (SEM, SEM, FEI Quanta-650 FEG, FEI Company, Hillsboro, OR, USA) equipped with an energy-dispersive spectrometer (EDS, EDAX-GENESIS, AMETEK, Inc., Berwyn, PA, USA). For the SEM and EDS observations, the samples were cut from the annealed plates, prepared via standard metallographic procedures, and etched with 4% nitric acid alcohol. The electron backscattered diffraction (EBSD) analysis was conducted with an acceleration voltage of 20 kV, samples tilt angle of 70°, and a working distance of 7 mm. An X-ray diffractometer (D/max-2500/PC, CuKα) was used to analyze the phase of the steel. X-ray diffraction (XRD, Rigaku Ultima IV, Akishima, Tokyo, Japan) measurements were conducted between 40 and 100° at room temperature using a diffractometer with a scanning rate of 1/min. Austenite content was determined by XRD based on the integrated intensities of (200)α, (211)α, (200)γ, (220)γ, and (311)γ diffraction peaks [20,21], and was calculated using Equation (1):

(1) $$V\gamma =1.4I\gamma /\left(I\alpha +1.4I\gamma \right)$$

where *Vγ* is the volume fraction of RA. *Iγ* is the integrated intensity of austenite and *Iα* is the integrated intensity of ferrite.

Samples of 6 mm width and 14 mm length for tensile tests were machined from the annealed sheets along the rolling direction. The uniaxial tensile testing was performed on an Instron electronic universal testing machine at room temperature with a strain rate of 5 × 10^−2^ s^−1^. The nanoindentation experiment uses Berkovich half-angle indenter, and the tip correction is conducted on fused silica. The loading time is 5 s, the retaining time is 2 s, the unloading time is 5 s, and the maximum loading load is 1000 μN. The indentation dot matrix of each polished sample is 5 × 6 dots.

## 3. Results and Discussion

### 3.1. Microstructure Evolution

**Figure 2:**
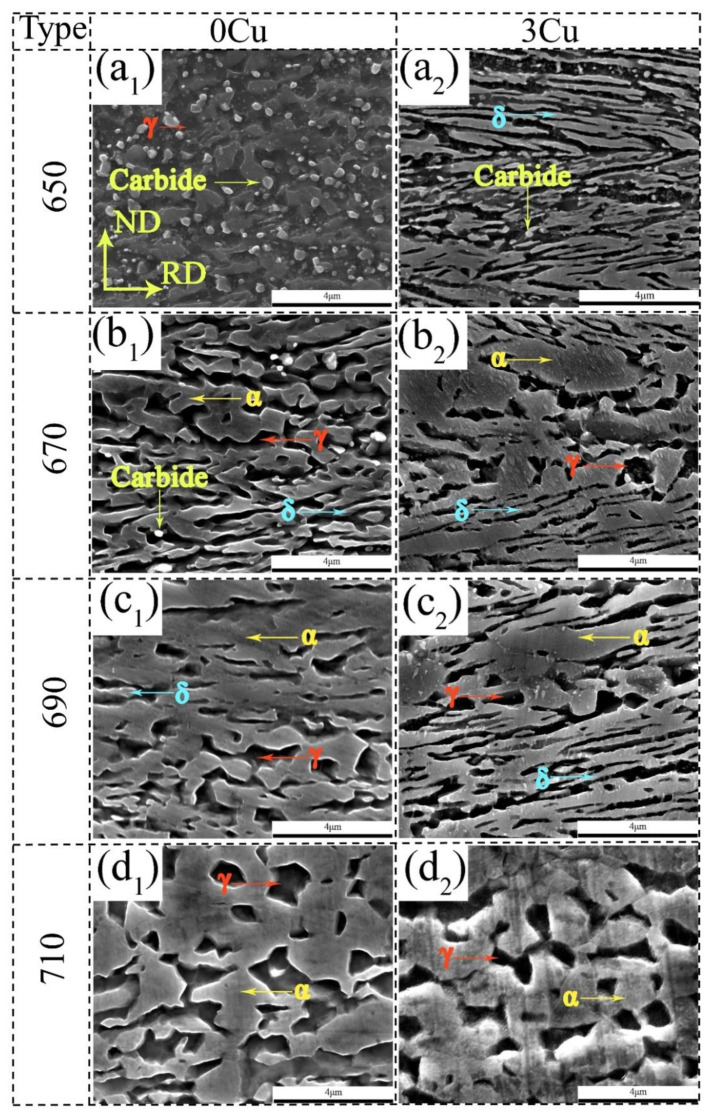
SEM micrographs at different annealing temperatures (**a_1_**) 0Cu650, (**b_1_**) 0Cu670, (**c_1_**) 0Cu690, (**d_1_**) 0Cu710, (**a_2_**) 3Cu650, (**b_2_**) 3Cu670, (**c_2_**) 3Cu690, and (**d_2_**) 3Cu710.

The SEM micrographs of the cold rolled steels annealed at 650, 670, 690, and 710 °C have been shown in Figure 2. The 0Cu650 and 3Cu650 sample showed a lath-like morphology of ferrite and austenite. White small carbides in the 0Cu650 sample still existed since the IA temperatures employed were substantially lower than the dissolution temperatures of possible carbides/precipitates; whereas most carbides dissolved in the 0Cu670 sample. As the annealing temperature increased to 690 °C, the 0Cu690, and 3Cu690 sample exhibited two different morphologies of ferrite and austenite i.e., lath and equiaxed. A minor amount of elongated δ-ferrite was noticeably present in the microstructure in these two samples. On increasing the IA temperature further to 710 °C, the 0Cu710 and 3Cu710 sample showed only equiaxed ferrite and austenite. 

**Figure 3:**
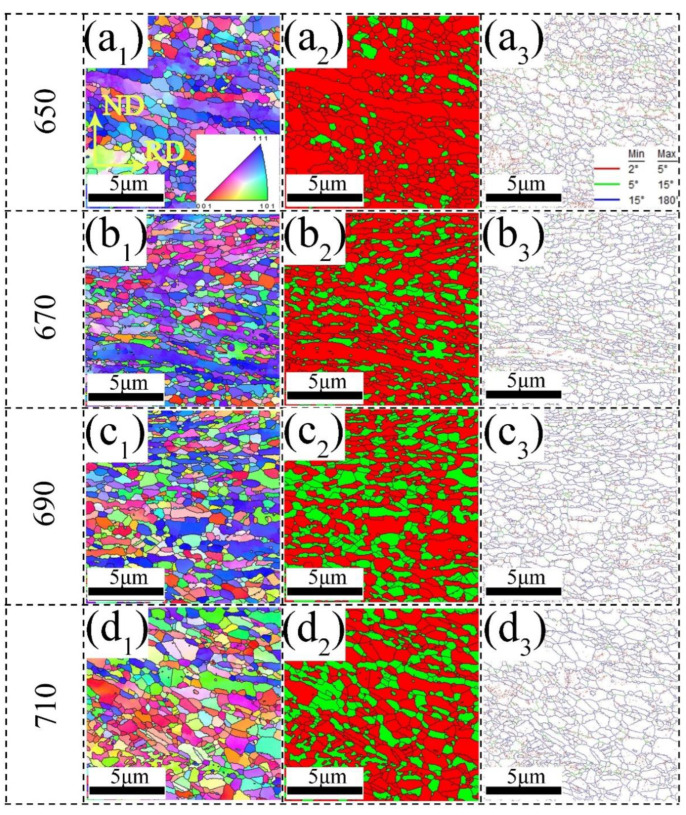
EBSD micrographs of 0Cu steel at different annealing temperatures (**a_1_**–**a_3_**) 650 °C, (**b_1_**–**b_3_**) 670 °C, (**c_1_**–**c_3_**) 690 °C, (**d_1_**–**d_3_**) 710 °C. (**a_1_**–**d_1_**) inverse pole figure (IPF) maps of both the bcc and fcc phases; (**a_2_**–**d_2_**) phase distribution map—RA as green and ferrite as red; (**a_3_**–**d_3_**) grain boundary maps, subgrain structures with 2–5° misorientation in red, low-angle grain boundaries with 5–15° in green, high-angle grain boundaries over 15° in blue. (For interpretation of the references to color in this figure legend, the reader is referred to the web version of this article).

**Figure 4:**
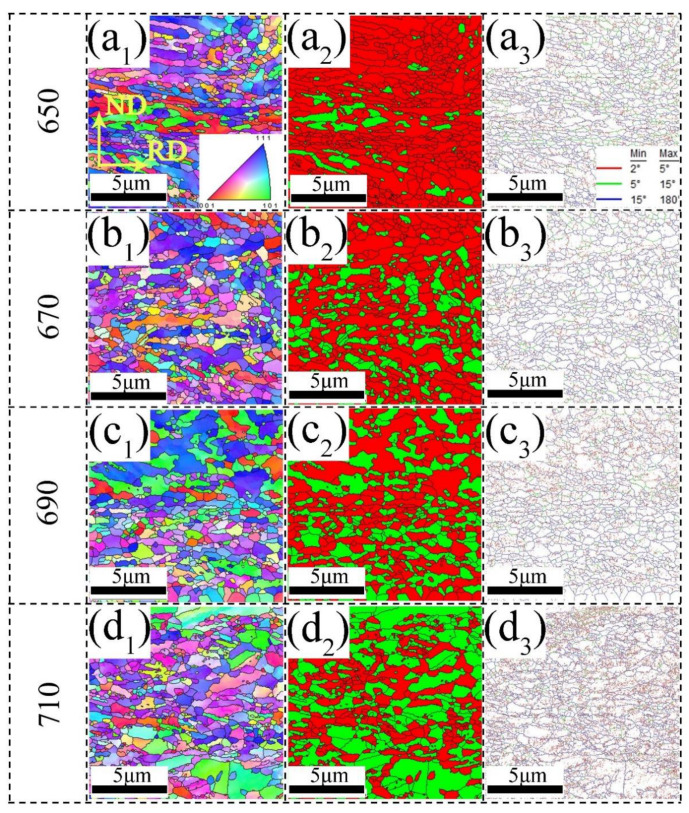
EBSD micrographs of 3Cu steel at different annealing temperatures (**a_1_**–**a_3_**) 650 °C, (**b_1_**–**b_3_**) 670 °C, (**c_1_**–**c_3_**) 690 °C, (**d_1_**–**d_3_**) 710 °C. (**a_1_**–**d_1_**) inverse pole figure (IPF) maps of both the bcc and fcc phases; (**a_2_**–**d_2_**) phase distribution map—RA as green and ferrite as red; (**a_3_**–**d_3_**) grain boundary maps, subgrain structures with 2–5° misorientation in red, low-angle grain boundaries with 5–15° in green, high-angle grain boundaries over 15° in blue. (For interpretation of the references to color in this figure legend, the reader is referred to the web version of this article).

To better understand the distribution of ferrite and RA of experimental steels, EBSD tests were conducted on both studied steels and the results are shown in Figure 3 and Figure 4, respectively. The annealed 0Cu steels have duplex microstructures of ferrite and austenite, as shown in Figure 3. Figure 3(a_1_–d_1_) reveal that there is a strong tendency for the <111> planes of the ferrite to align in the steel sheets. This ferritic texture may be inherited from the cold-rolled martensite, which was subsequently subjected to IA [22]. The overall volume fraction and grain size of austenite in the 0Cu650 sample are 10.2% and 0.32 μm, respectively. As the IA temperature increased to 670 °C, the austenite grain size increased up to 0.34 μm, while its volume fraction was 25.4%. In the 0Cu690 steel, both the grain size and volume fraction of austenite grain increase to 0.37 μm and 41%, respectively. With the IA temperature further increasing to 710 °C, the volume fraction and grain size of austenite are 55% and 0.47 μm, respectively. As the IA temperature increases from 650 °C to 710 °C, the fraction of high angle grain boundaries (HAGBs) is 50%, 65.7%, 72%, and 74.6%, respectively. 

Similar to the annealed 0Cu steels, the 3Cu steels consist of duplex microstructures of ferrite and austenite, as shown in Figure 4. As presented in Figure 4(a_1_–d_1_), the <111> planes of the ferrite and <101> planes of austenite have a strong tendency to align in the steel sheets. For the 3Cu650 sample, the average grain size and volume fraction of austenite are 0.32 μm and 10.5%, respectively. With the IA temperature increasing to 670 °C, the average grain size and volume fraction of austenite are 0.35 μm and 28%, respectively. As the IA temperature increases to 690 °C, the austenite grain size increases up to 0.39 μm, while its volume fraction is 44%. For the 3Cu710 specimen, both the grain size and volume fraction of austenite grain increase to 0.47 μm and 57.8%, respectively. The fraction of HAGBs of the 3Cu steels is 48.8%, 62.2%, 70%, and 72.3%, respectively, with the IA temperature increasing. 

### 3.2. Mechanical Properties

**Figure 5:**
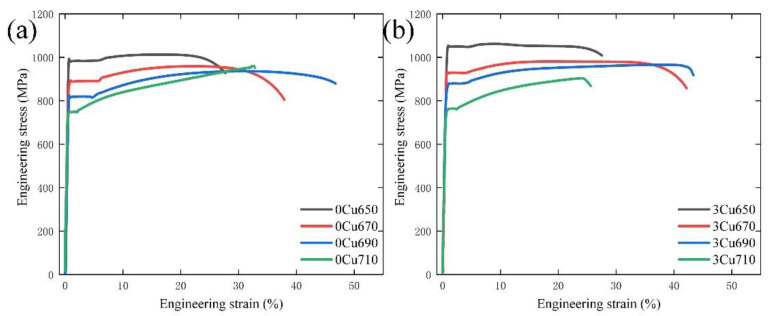
Engineering stress-strain curves of (**a**) 0Cu steel and (**b**) 3Cu steel.

The room temperature engineering stress-strain curves of two experimental steels are presented in Figure 5, and the detailed mechanical properties are summarized in Table 2. The yield strength (YS) and UTS of the 0Cu650 are 992 MPa and 1012 MPa, respectively. As the IA temperature decreased, the YS and UTS decreased to 892 MPa and 959 MPa, and 824 MPa and 936 MPa in the 0Cu670 and 0Cu690 specimens, respectively. With increasing IA temperature from 650 °C to 690 °C, the TE increased from 27.6% to 44.5%. As IA temperature increased to 710 °C, the YS, UTS, and TE were 750 MPa, 960 MPa, and 32.7%, respectively. Due to the high IA temperature, the average grain size of the sample annealed at 710 °C is larger than other samples, leading to the lowest YS of experimental steels. At higher IA temperature, the austenite is unstable and quickly transform to martensite during deformation, resulting in low TE. The evolution of mechanical properties of 3Cu steel with IA temperature is similar to that of 0Cu steel. The PSE of both experimental steels increases with increasing IA temperature and then decreases further with further annealing. After intercritically annealed at 690 °C for 1 h, the PSE of 0Cu and 3Cu steels is 41.6 GPa% and 43.5 GPa%, respectively, which are higher than other samples subjected to different IA temperatures. From Table 2, it can be noted that the Lüders bands strain (LBS) decreases with IA temperature increasing, which is also founded in other medium-Mn steels [23,24]. Notably, the magnitude of LBS in the 3Cu steel is lower than that of 0Cu steel. Thus, the Cu addition not only increases the YS of medium Mn steel but also benefits the decrease of LBS. 

**Figure 6:**
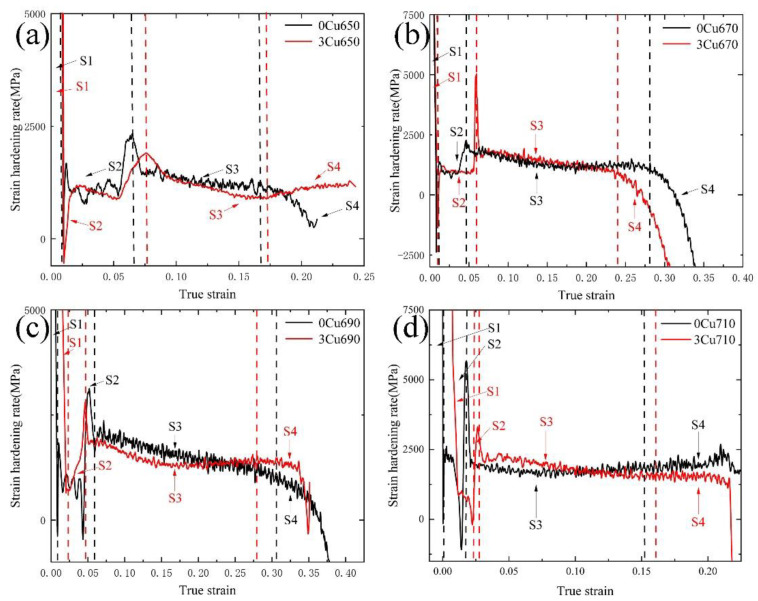
Strain hardening curves of experimental steels at different annealing temperatures (**a**) 650 °C, (**b**) 670 °C, (**c**) 690 °C, (**d**) 710 °C.

Figure 6 presents the change in strain hardening rate (SHR) with true strain in the experimental steels intercritically annealed at temperatures ranging from 650 °C to 710 °C. The samples of two experimental steels at the same IA temperature exhibit similar SHR curves. The SHR curves can be divided into four stages, as shown in Figure 6. In stage I (S1), the SHR curves exhibit a rapid decrease, most likely due to the dynamic recovery of dislocations [14]. With true strain further increasing, the SHR curves are relatively steady at the beginning of stage 2 (S2) and increased significantly due to the end of propagation of Lüders bands. In stage 3 (S3), the SHR curves decrease gradually with the true strain increasing. Finally, in stage 4 (S4), as the true strain increases, the SHR curves decrease dramatically until the specimens fail. 

### 3.3. Fracture Morphology

**Figure 7:**
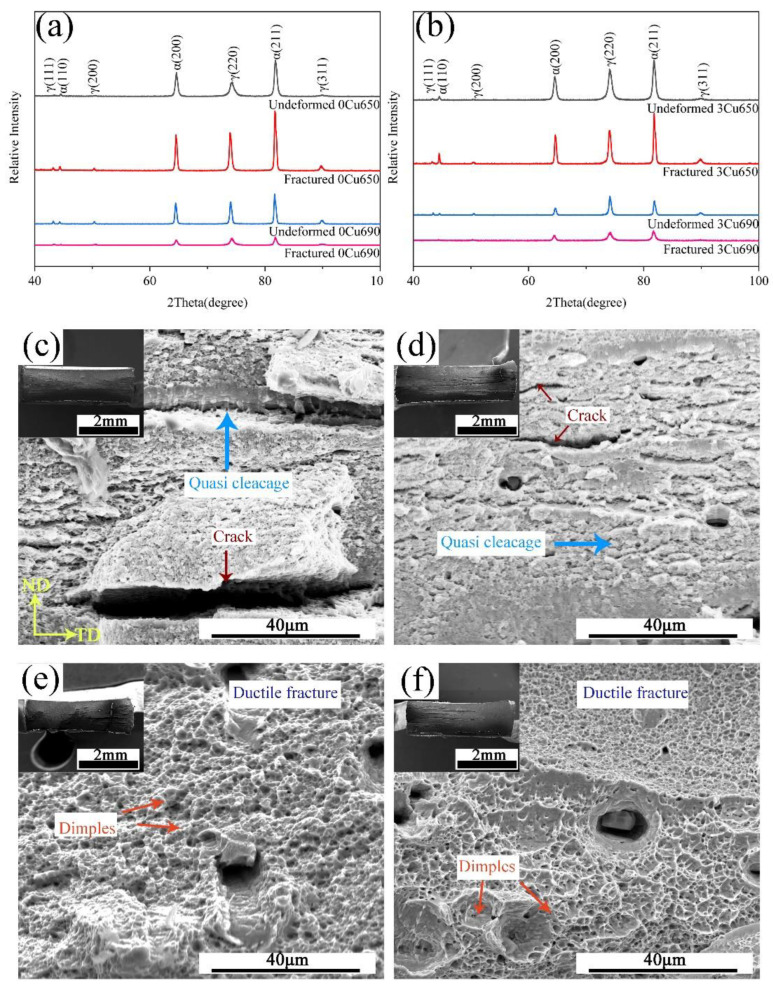
XRD patterns of (**a**) 0Cu650 sample and 0Cu690 sample and (**b**) 3Cu650 sample and 3Cu690 sample. Fracture morphology of the experimental steels (**c**) 0Cu650, (**d**) 3Cu650, (**e**) 0Cu690, (**f**) 3Cu690.

Figure 7a,b show the XRD patterns of undeformed and fractured 0Cu and 3Cu steels after IA at 650 °C and 690 °C, respectively. For the 0Cu650 and 3Cu650 samples, the volume fraction of austenite is low and the austenite is quite stable. No obvious TRIP effect occurred during tensile deformation. After the samples fracture, the microstructure still consists of about 10% austenite. As the IA temperature increased, the volume fraction of austenite in the 0Cu690 and 3Cu690 samples was 26.2% and 28.7%, respectively. Moderate austenite stability in these two samples contributes to the large TE. The fracture morphology of the studied steels annealed at 650 °C and 690 °C are shown in Figure 7b,d. It can be seen that the fracture morphology of 0Cu650 and 3Cu650 is a quasi-cleavage fracture. For these two samples, a very limited amount of austenite transformed into martensite because of high stability. Cracks could be observed in Figure 7b,c, which were caused by the numerous voids interconnection. The fracture behavior of the studied steels was just like that of ultrafine-grained metallic materials, resulting in a brittle fracture manner. With IA temperature increasing to 690 °C, the 0Cu690 and 3Cu690 samples had a clear ductile region with large and deep dimples, as shown in Figure 7e,f. There is a small brittle region in 0Cu690 and 3Cu690 samples featuring the crack in the second hard phase particles. The ductile fracture in the 0Cu690 and 3Cu690 samples is closely related to the microstructure consisting of a high volume fraction of austenite with moderate stability. 

## 4. Discussion

**Figure 8:**
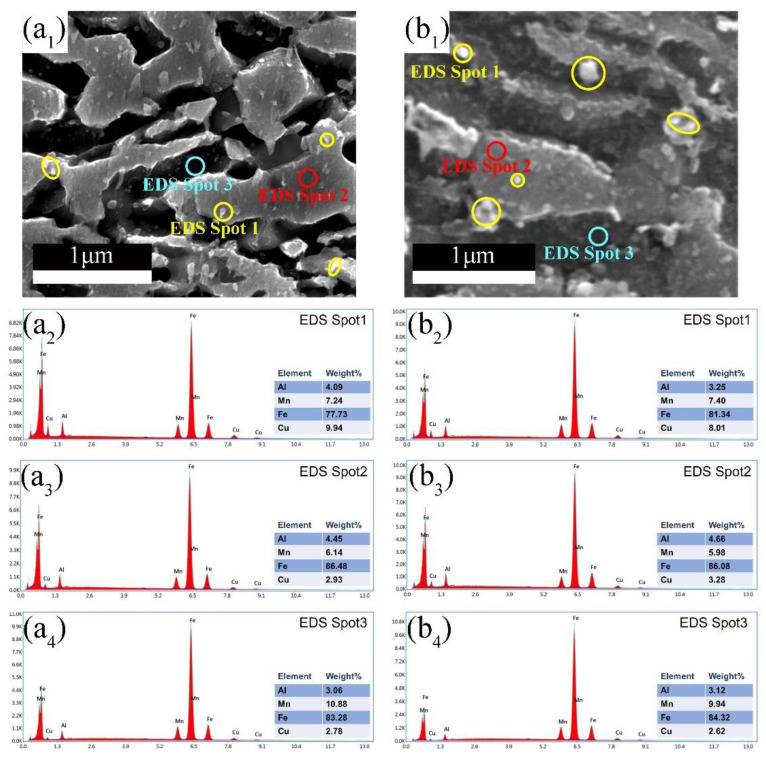
Chemical composition of Cu-rich nanoparticle, ferrite and austenite measured through EDS in 3Cu steel (**a_1_**–**a_4_**) 3Cu670 sample; (**b_1_**–**b_4_**) 3Cu690 sample. (**a_1_**–**b_1_**) micrograph showing location of points within phases on which EDS was conducted; (**a_2_**–**b_2_**), (**a_3_**–**b_3_**) and (**a_4_**–**b_4_**) showing representative EDS spectrum from Cu-rich nanoparticle, ferrite, and austenite, respectively.

In this study, 3 wt% of Cu was added into Fe-0.4C-7Mn-4Al steel having a ferrite and austenite duplex microstructure. The effects of Cu addition on the microstructure and mechanical properties are discussed as follows. Effects of the Cu addition on tensile properties are more influenced by the microstructural variation [17]. As a weak austenite stabilizer, the addition of Cu slightly raises the volume fraction of austenite. At the IA temperature of 670 °C, the addition of Cu raises the volume fraction of RA from 25.4% to 28%, and the raised RA fraction is 3% and 2.8% in the samples subjected to IA temperature of 690 °C and 710 °C, respectively. In other medium Mn steels, the addition of Cu may lead to the Cu-rich particles precipitating in the matrix [17,18]. To clarify composition partitioning between ferrite and austenite, point EDS was conducted on sample 3Cu670 and 3Cu690, as shown in Figure 8. It is noted that Mn is enriched in austenite, while the enrichment of Al in ferrite is distinct. In addition, there are some nanoscale Cu-rich particles dispersed in the ferrite-austenite interfaces and ferrite matrix in the samples, which are marked by yellow circles as shown in Figure 8(a_1_,b_1_). Moreover, another effect of the Cu addition is the delay of recrystallization, which is evidenced in the fraction of HAGBs in 3Cu steel is lower than that in 0Cu steel at the same IA temperature. Because of a pinning or solute drag effect, solute atoms or precipitates can delay the degree of recrystallization by influencing the movement of dislocations or grain boundaries [25,26,27]. Song et al. [17] demonstrated the addition of Cu in Fe-0.5C-12Mn-7Al steel delays the recrystallization by solute atoms or precipitates. Further in-depth investigation of Cu-rich nanoparticles and the influences of Cu addition on the Lüders bands in medium Mn steels will be covered in our ongoing research. 

**Figure 9:**
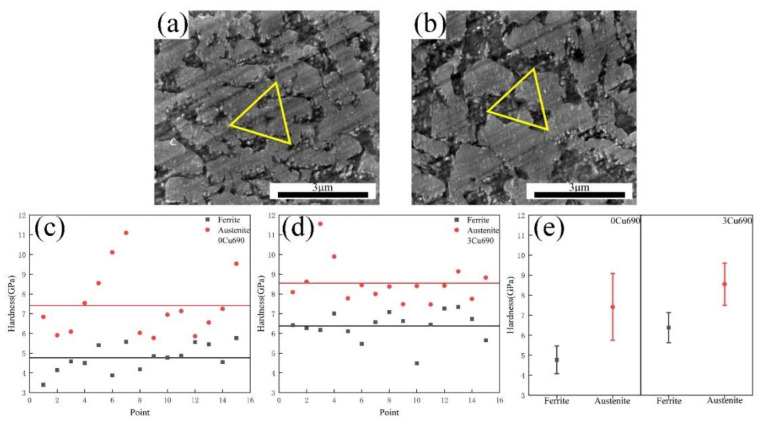
SEM image showing the indentation impression on (**a**) ferrite grain and (**b**) austenite grain. Triangles mark the position of indentation impressions. Hardness distribution of austenite grains and ferrite grains (**c**) 0Cu690 sample; (**d**) 3Cu690 sample; (**e**) average hardness of ferrite and austenite grains in 0Cu690 sample and 3Cu690 sample.

The obvious effect of the Cu addition on the mechanical properties is the increase of YS of experimental steels. As the IA temperature increased from 650 °C to 710 °C, the YS of 3Cu steel increased by 60 MPa, 40 MPa, 55 MPa, and 12 MPa, respectively, compared to that of 0Cu steel at the same IA temperature. The strengthening effect of Cu addition is further analyzed via a nanoindentation experiment. According to the SEM micrograph of samples after the nanoindentation experiment, the positions of indents can be determined. It can be noted that the indent in Figure 9a is on the ferrite, while the indent in Figure 9b is on the austenite. From Figure 9c–e, it can be seen that both the ferrite and austenite of the 3Cu690 sample are harder than those of the 0Cu690 sample. The average hardness of the ferrite and austenite in the 0Cu690 sample is 4.7 GPa and 7.4 GPa, respectively. With the Cu addition, the average hardness of the ferrite and austenite in the 3Cu690 sample increases to 6.3 GPa and 8.5 GPa, respectively. The hardness difference of the ferrite between the 3Cu690 and 0Cu690 samples (6.3 GPa vs. 4.7 GPa) is higher than that of the austenite (8.5 GPa vs. 7.4 GPa). The hardness difference in the ferrite likely corresponds to Cu-rich nanoparticles formed in the ferrite. In contrast, the hardness difference in austenite may be attributed to the effect of solid solution hardening due to the Cu being an austenite stabilizer [17]. According to Rodríguez et al. [28], the hardness values obtained from the nano-indentation test were linearly proportional to YS obtained from tensile tests and the proportional constants of YS were 4.15. Thus, the Cu addition increases the YS of experimental steels by 57.3 MPa via using the rule of mixtures, considering the volume fraction of the constituent phases as mentioned in Section 3.1. This result is very close to the increase of YS of 55 MPa obtained from stress-strain curves.

The hardness of austenite in both 0Cu690 and 3Cu690 is higher than that of ferrite as mentioned before, which may be because of the higher working hardening rate of austenite. It should be noted that a large scatter in hardness in austenite is observed, while the variation in hardness in ferrite is slight. Thus, it is necessary to investigate load-displacement (*P-h*) curves of both austenite and ferrite since these two phases also demonstrate elastic-plastic deformation during the nanoindentation tests [29,30]. The elastic deformation in austenite and ferrite is investigated via the Hertzian elastic contact solution as below [31]:

(2) $$P=\frac{4}{3}E\gamma R_{i}^{\frac{1}{2}}h^{\frac{3}{2}}$$

and

(3) $$\frac{1}{E_{\gamma }^{2}}=\frac{1-v_{i}^{2}}{E_{i}^{2}}+\frac{1-v_{s}^{2}}{E_{S}^{2}}$$

where *R*_i_ = 200 nm is the indenter tip radius. *E_r_* is the effective indentation modulus, *E* is Young’s modulus, *v* is Poisson’s ratio, and the subscripts *i* and *s* represent the indenter and specimen, respectively [32]. The Young’s modulus and Poisson’s ratio for the indenter, ferrite phase, and austenite phase are 1141 GPa, 0.07 [32], 196 GPa, 0.28 [33], and 186 GPa, 0.24 [33], respectively. 

The plastic deformation of austenite and ferrite phases of experimental steels during nanoindentation test can be studied through the concept of geometrically necessary dislocation (GND) as follows [29,34]:

(4) $$P=Ac\mathrm{MC}\beta Gb\sqrt{\rho \mathrm{SSD}+\rho \mathrm{GND}}$$

and

(5) $$\rho_{GND}=\frac{3}{2}\frac{1}{f^{2}}\frac{\tan^{2}\theta }{bh}$$

where *P* is the applied load. *A_c_* = 24.5 *h*^2^ is the projected area for an ideal Berkovich indenter, *M* = 3 is the Taylor factor, *C* = 3 is the constraint factor, *β* = 0.5 is a parameter representing the dislocation structure, *b* = 0.25 nm is the Burgers vector for both ferrite and austenite, *θ* is the angle between the surface and the indenter, *f* is the ratio of the plastic zone radius *a*_pz_ to the contact radius *a*_c_, *ρ_GND_* is the GND density in the plastic zone [32]. Since the small indentation depth, the statistically stored dislocation density *ρ_SSD_* in the plastic zone is not considered here. *G* is the shear modulus which can be calculated from Young’s modulus and Poisson’s ratio [29].

**Figure 10:**
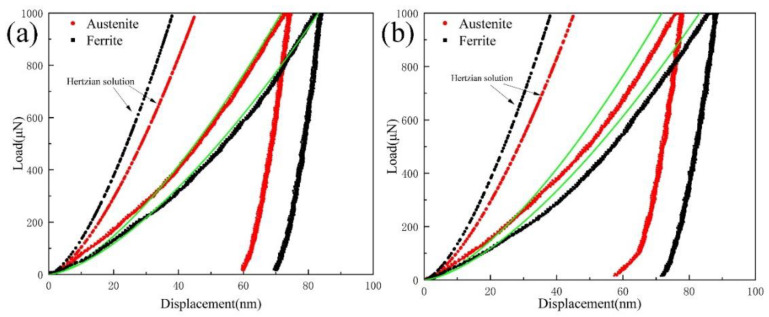
*P-h* curves of indentations on (**a**) 0Cu690 and (**b**) 3Cu690. The dashed line is the Hertzian solution. The solid green line represents the fitting by using the GND concept. (For interpretation of the references to color in this figure legend, the reader is referred to the web version of this article).

As shown in Figure 10, the elastic-plastic behavior during the nanoindentation test could be separated according to the fitting by the Hertzian elastic contact solution and GND concept. The maximum shear stress (*τ*_max_) beneath the indenter can be calculated as [31]:

(6) $$\tau_{\max}=\frac{0.37P^{\frac{1}{3}}E_{r}^{\frac{2}{3}}}{\pi R_{i}^{\frac{2}{3}}}$$

*τ*_max_ values of all phases in experimental steels were calculated to be in the range of 5.6–8.2 GPa, which is within the theoretical strength of crystalline materials by considering the shear modulus of steels as about 80 GPa at room temperature [32]. These results indicate that the elastoplastic transition in the specimens is the result of dislocation nucleation rather than the movement of the existing dislocation [29]. It should be noted that the austenite in the 0Cu690 and 3Cu690 steel transforms into martensite during tensile deformation. During the nanoindentation tests, some austenite may transform to martensite. The transformed martensite close to the indenter may deform plastically during the indentation, contributing to the increase in the overall hardness. Therefore, it could be reasonable to select the hardness of austenite above 10 GPa to represent the formation of martensitic transformation in the *P-h* curves. 

## 5. Conclusions

In the present study, high-strength Cu-bearing medium-Mn duplex ferrite-austenite steels (Fe-0.4C-7Mn-4Al-0/3Cu wt%) were developed, and the microstructural evolution and mechanical properties with IA temperature changing were investigated. Conclusions can be summarized as follows:

(1) As the IA temperature increased from 650 °C to 710 °C, the volume fraction of austenite of 0Cu steel and 3Cu steel increased from 10.2% to 55% and 10.5% to 57.8%, respectively. At a given IA temperature, the Cu addition slightly raised the volume fraction of austenite. The average grain size of austenite and fraction of HAGBs in both experimental steels increased with IA temperature increasing. 

(2) With increasing annealing temperature, the YS, UTS, and LBS decreased in both experimental steels. The Cu addition not only increases the YS of medium Mn steel but also facilitates the decrease of LBS. The TE and PSE increase with increasing IA temperature and then decrease further with further annealing. After intercritically annealed at 690 °C, the 3Cu steel demonstrates the best comprehensive mechanical properties of 43.5 GPa%. 

(3) Cu-rich nanoparticles were found precipitated in the 3Cu steels. The strengthening effect of the Cu addition was evidenced by the nanoindentation tests. The average hardness of austenite and ferrite in steel without Cu addition is 7.4 and 4.7 GPa, respectively. The addition of Cu raised the average hardness of retained austenite and ferrite to 8.5 and 6.3 GPa, respectively. 

## Figures and Tables

**Table 1:** Chemical composition of the experimental steels (wt%).

	Mn	Al	C	Cu	S	P	Fe
0Cu	7.11	3.85	0.397	0	0.0075	0.0082	Bal
3Cu	6.94	3.86	0.387	3.04	0.0073	0.0081	Bal

**Table 2:** Comprehensive mechanical properties of samples at room temperature after annealing at different temperatures.

Sample	YS (MPa)	TS (MPa)	TEL (%)	LBS (%)	PSE (GPa%)
0Cu650	992	1012	27.6	5.3	27.9
0Cu670	892	959	37.9	5	36.3
0Cu690	824	936	44.5	3.8	41.6
0Cu710	750	960	32.7	1.3	31.4
3Cu650	1052	1028	27.5	3.5	28.2
3Cu670	932	980	42.1	2.9	41.3
3Cu690	879	966	45	2.5	43.5
3Cu710	762	900	27.2	1.0	24.5

## Data Availability

Not applicable.
